# Estimation of tumor heterogeneity using CGH array data

**DOI:** 10.1186/1471-2105-10-12

**Published:** 2009-01-09

**Authors:** Kai Wang, Jian Li, Shengting Li, Lars Bolund, Carsten Wiuf

**Affiliations:** 1 Institute of Human Genetics, University of Aarhus, Aarhus, Denmark; 2 BiRC – Bioinformatics Research Center, University of Aarhus, Aarhus, Denmark

## Abstract

**Background:**

Array-based comparative genomic hybridization (CGH) is a commonly-used approach to detect DNA copy number variation in whole genome-wide screens. Several statistical methods have been proposed to define genomic segments with different copy numbers in cancer tumors. However, most tumors are heterogeneous and show variation in DNA copy numbers across tumor cells. The challenge is to reveal the copy number profiles of the subpopulations in a tumor and to estimate the percentage of each subpopulation.

**Results:**

We describe a relation between experimental data and exact DNA copy number and develop a statistical method to reveal the heterogeneity of tumors containing a mixture of different-stage cells. Furthermore, we validate the method on simulated data and apply the method to 29 pairs of breast primary tumors and their matched lymph node metastases.

**Conclusion:**

We demonstrate a new method for CGH array analysis that allows a tumor sample to be classified according to its heterogeneity. The method gives an interpretable series of copy number profiles, one for each major subpopulation in a tumor. The profiles facilitate identification of copy number alterations in cancer development.

## Background

Heterogeneity is an important characteristic of most cancers. It manifests itself in various different ways, for example as heterogeneity in gene expression, protein abundance and/or genomic DNA copy number [1-3]. In this paper we focus exclusively on heterogeneity in genomic DNA copy number. Genomic DNA copy number variation in a tumor reflects concomitant or successive development of various foci and indicates that malignant transformation of cells is a dynamic evolutionary process. Numerous studies have demonstrated that the development of tumors involves accumulation of various genetic alterations [4-8]. Comparative genomic hybridization (CGH), matrix-based BAC/oligo array CGH, or oligonucleotide-based arrays are techniques that frequently are applied to elucidate *intertumor *heterogeneity across cancers, patients or stages; the genomic profile of a tumor is presented at a fixed time point and averaged across different cells in the tumor.

In contrast, *intratumor *heterogeneity is rarely reported [9]. Laser-capture micro-dissection is a powerful tool to select few phenotypically homogeneous tumor cells, and thus a way circumvent the problem of averaging across many potentially inhomogeneous tumor cells. Methods for whole genome amplification enable researchers to obtain sufficient DNA for CGH analysis even from few cells [10]. In this way the genomic profile of a small (homogeneous) region in the tumor can be studied, whereas the heterogeneity of the tumor might be elucidated by investigating several different regions across the tumor. Naturally, the latter is time-consuming and labor intensive, and to our knowledge, has not been reported.

With the above in mind, we have developed a statistical method to study tumor heterogeneity. It takes CGH array data from individual tumors as input; one tumor sample is represented by one array and contains DNA from a potentially heterogeneous cell population. Our method estimates the number of dominant tumor subpopulations, the percentages of the subpopulations in the sample, and the copy number profiles of the dominant subpopulations. Also, the method estimates the percentage of normal cells. Normal cells are diploid (two copies of all genomic DNA) and typically consist of nonmalignant epithelium, fibroblast and/or penetrated lymphocytes. To validate the method we have simulated data according to a model derived from real CGH data. Additionally, we have mixed some real tumor samples to obtain samples with partially known profiles. Subsequently, we applied our method to 29 paired primary and lymph node metastasis breast cancer samples.

Our method can be considered a classifier in the sense that it assigns a number of subpopulations to a given tumor sample. Alternatively the method might be considered as a model selection procedure over an extensive number of models: We seek the model that explains the data best, optimizing over the number of subpopulations and the copy number profile for each subpopulation.

## Results and discussion

### Calibration experiment

A series of calibration experiments were conducted to test the array CGH platform in our laboratory. The majority of samples were from normal males and females (diploid samples), but some samples were from patients with genomic abnormalities, e.g. trisomies and monosomies. Importantly, all these samples are assumed to be homogeneous, i.e. all cells in a sample have the same copy number alteration(s). We fit a linear model to describe the relationship between log-copy number and log-intensity; as described in **Methods (The copy number model)**. The parameters of the linear model, *y *= *αx *+ *β*, are estimated to $\hat{\alpha }$ = 0.6049, 95% CI: (0.5542,0.6556), and $\hat{\beta }$ = -0.039, 95% CI (-0.085,0.0067), respectively. The observed and fitted values show high correlation; Pearson's regression coefficient *R*^2 ^is greater than 0.98. The observed values and the regression line are shown [see Additional file 1].

### Heterogeneity in real tumors

We applied the procedure described in **Methods (Classification of samples) **to estimate the level of genomic heterogeneity in 29 pairs of primary tumor and lymph node metastasis.

The method allows us to estimate the number of dominant subpopulations, the copy number profile and the percentage of cells in each subpopulation. Our method assumes a model of sequential tumor evolution where each subpopulation is evolved from the previous population by introducing new aberrations, or by making aberrations in the previous population more extreme, i.e. by increasing copy numbers or decreasing copy numbers, see **Methods (Mixture modeling of tumor samples) **for details.

The results from the analysis of the primary tumor and lymph node metastasis are shown in details [see Additional file 2] and summarized in Table 1. To estimate the complexity of a tumor, we introduce the following measure called the Aberration Index (AI),

**Table 1:** Subpopulation summary of the 29 pairs of primary and metastasis samples

	#	*P*_1_	*P*_2_	*P*_3_	*AI*_1_	*AI*_2_	*AI*_3_	Total	Pure
T-2	15	25.3 (8.8)	-	-	1.13 (0.76)	-	-	0.25 (0.13)	1.13 (0.76)
T-3	13	33.4 (9.4)	14.2 (5.1)	-	0.34 (0.13)	1.18 (0.26)	-	0.28 (0.10)	0.59 (0.20)
T-4	1	23 (-)	34 (-)	8 (-)	0.25 (-)	0.53 (-)	1.40 (-)	0.35 (-)	0.54 (-)
M-2	16	24.8 (8.3)	-	-	1.07 (1.00)	-	-	0.21 (0.11)	1.07 (1.00)
M-3	10	32.5 (8.7)	15.1 (6.1)	-	0.45 (0.23)	1.33 (0.41)	-	0.33 (0.12)	0.71 (0.30)
M-4	3	16.7 (3.1)	31 (6)	8.7 (2.1)	0.18 (0.04)	0.48 (0.11)	1.33 (0.17)	0.29 (0.05)	0.52 (0.09)

The table summarizes the analysis done on the 29 pairs of samples [see Additional file 2]. Shown are mean values with standard deviation in parenthesis. T-*i*: Primary tumor with *i *subpopulations, M-*i*: Metastasis with *i *subpopulations, #: Number of samples, *p*_*k*_: Percentage of (abnormal) subpopulation *k *≥ 1, *AI*_*k*_: Aberration Index for subpopulation *k*, Total: Weighted sum of *AI*_*k*_, Σ_*k*_*p*_*k*_*AI*_*k*_, Pure: *AI*_•_, normalized weighted sum of *AI*_*k*_, *cf*. equation (2).

(1) $$AI_{k}=\frac{\sum_{i}m_{i}|C_{ik}-2|}{\sum_{i}m_{i}},$$

where *m*_*i *_is the number of clones in segment *i*, *C*_*ik *_the copy number of segment *i *in subpopulation *k*, and |*x*| denotes the absolute value of *x*. Here we assume clones are uniformly spaced across the genome; if this is not the case the contribution from each clone can be weighted by its distance to neighbor clones. The estimated subpopulations are named P0, P1, P2, ..., and ordered according to increasing AI. P0 consists of normal cells only and has *AI*_0 _= 0.

Tumors with only one abnormal subpopulation have higher average AI than tumors with many abnormal subpopulations; see Table 1. Also, the average complexity of all abnormal subpopulations in a tumor,

(2) $$AI_{•}=\sum_{k>0}\frac{p_{k}AI_{k}}{1-p_{0}},$$

("Pure" in Table 1) where *p*_*k *_denotes the percentage of subpopulation *k*, is decreasing with the number of subpopulations. This is re ecting that the percentage of the most complex subpopulation is generally not very high, whereas the percentage of the least complex is relatively much higher.

**Figure 1:**
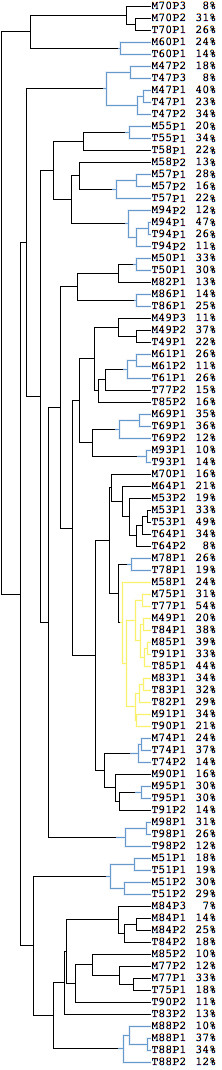
**Cluster diagram of the 29 pairs of tumors**. The 29 pairs of primary and metastasis samples were divided into 89 subpopulations using the method described in the paper. For two leaves with the same ID (e.g. T53), P1 refers to the abnormal subpopulation with the least aberration, P2 (if it exists) refers to the subpopulation with more aberrations than P1, and P3 (if it exists) the one with most aberrations. The percentage of each subpopulation is also included. The cluster diagram was generated using average linkage clustering based on the estimated copy numbers for all 3340 clones. Reducing the number of clones produces very similar results.

We clustered all subpopulation profiles, rather than just the overall profiles of the samples. The result, shown in Figure 1, presents the similarity among all subpopulations across the 29 tumor pairs. In 16 cases (out of 29) all primary and metastasis subpopulations cluster together (shown in blue in the figure), i.e. the subpopulations of the metastasis are more similar to the subpopulations of the primary tumor than to subpopulations of other samples. The high similarity between the primary tumor and the lymph node metastasis from the same patient indicates that biological characteristics of the primary tumor are maintained in the lymph node metastasis. In other samples the primary tumor and the metastasis show much less similarity, and even within a sample, the subpopulations can be very dissimilar. The yellow cluster in Figure 1 consists mainly of subpopulations with low AI, i.e. few genomic aberrations.

Table 2 shows the relationship between the estimated number of subpopulations in the primary tumor and in the corresponding metastasis. There is not a clear relationship between the two numbers with only 14 pairs out of 29 showing the same subpopulation number. Of the 16 tumor pairs that clustered together in Figure 1 (see above paragraph), 10 pairs have the same subpopulation number while in 4 cases the metastasis shows a lower number than the primary tumor.

**Table 2:** Primary tumor *vs. metastasis*

	Metastasis		
Primary	2	3	4
2	9	4	2
3	7	5	1
4	0	1	0

Shown is the estimated number of subpopulations in the primary tumor and the corresponding lymph node metastasis.

The average percentage of the normal cell subpopulation in a tumor is around 60%, which is higher than we expected. According to the pathologists involved in removing the tumors by surgery, the samples contain at least 70% malignant cells. However, this percentage is judged by eye and represents how big a fraction of the tumor that appears to consist of normal cells. This is likely to be an overestimate because malignant cells generally are bigger than normal cells [11]. Also, some normal cells are typically removed before the samples are subjected to array analysis.

**Figure 2:**
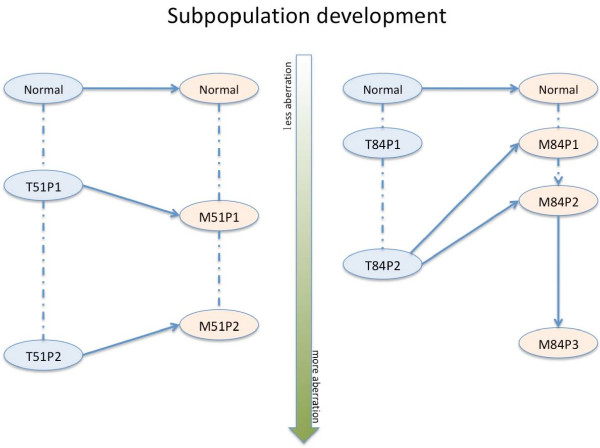
**Subpopulation development**. Here we show possible subpopulation development in two paired tumor samples. The dashed lines connect the subpopulations from same sample. The solid and dashed lines represent the most likely and the least likely development path, respectively. From the top-down, the subpopulation contains more and more genomic aberrations.

Finally, based on our results, we can make some predictions about possible subpopulation developments in paired tumor samples (Figure 2). For example, the P1 (P2) subpopulations from the samples T51 and M51 cluster together and they are likely immigrated from the primary tumor to the lymph node as a whole. Whereas, for example the subpopulations of M84 do not cluster together with T84-P1, which might indicate that the metastasis has arisen from the T84-P2 in the primary tumor.

### Simulation results

We simulated samples based on the 29 pairs of real samples (in total 58 samples), as described in the **Methods (Simulation)**. For each real sample we fit copy number profiles assuming 2, 3 or 4 subpopulations and use these profiles as templates for simulation of artificial log-intensities. For example, for a real sample with an estimated 2 subpopulations, we fit 2, 3 and 4 subpopulations and simulate log-intensities based on these profiles. In this case, the profiles of the four subpopulations are very similar because the real sample is best explained by two subpopulations. Subsequently, we applied our method to the simulated samples; the results are summarized in Tables 2 and 3.

**Table 3:** Prediction accuracy for simulated samples

	Simulated	Predicted as			Correct
Real		2	3	4	(in %)
2	2	115	9	0	0.93
	3	61	59	4	0.48
	4	57	48	19	0.15

3	2	80	11	1	0.87
	3	8	78	6	0.85
	4	6	60	26	0.28

4	2	15	1	0	0.94
	3	0	13	3	0.81
	4	0	1	15	0.94

For each real sample four simulated samples were created; in total 174 samples. The real sample was used as template for the simulated samples. In the table, the simulation results are shown according to the estimated number of subpopulations in the real samples. Real: Estimated number of subpopulations in the real sample, Simulated: The number of subpopulations in the simulated sample, Predicted: The predicted number of subpopulations in the simulated sample.

We learn several things from the simulations (Table 3). Generally, it is possible to predict the number of subpopulations under the chosen simulation model. However, samples with 3 or 4 similar subpopulations (Table 3: Real = 2, Simulated = 3 or 4) are difficult to predict correctly (48% and 15%, respectively), whereas samples with few less similar subpopulations are much easier to predict; in Table 3 simulated samples with 2 subpopulations achieve a prediction accuracy above 87%.

Next, we estimated the accuracy of the predicted subpopulation percentages and the corresponding profiles (Table 4). When the subpopulation number was predicted correctly, the estimated subpopulation percentages and copy number profiles were compared to the values used in the simulation. Table 4 shows that the copy numbers of the abnormal subpopulations are predicted correctly for more than 80% of the clones, with higher accuracy obtained when there are few subpopulations than many.

**Table 4:** Accuracy of copy numbers and percentages

	#Subpopulations		
	2	3	4
A (in %)	2.05 (2.26)	2.76 (2.50)	4.78 (4.01)
B (in %)	89.5 (13.6)	82.8 (10.0)	80.5 (9.0)

The table shows accuracy of the estimated copy numbers and subpopulation percentages when the number of subpopulations is correctly predicted. A) The average absolute difference between the estimated and true percentages in the simulated samples, B) The average number of times the copy number was predicted correctly, excluding the normal subpopulation. Standard deviations in parenthesis.

Our method of estimation assumes a model of sequential tumor evolution. To test the method's robustness to violations of the model we did the following; see **Methods (Simulation) **for details. First we simulated a sample according to the model. Then, for each segment we modified the copy numbers by adding/subtracting a Poisson number of copies. The results are shown in Table 5. Even when the percentage of segments violating the model is high, we predict the correct subpopulation number in the majority of the cases.

**Table 5:** Robustness of the method

	3 subpopulations			4 subpopulations		
*λ*	1	0.5	0.25	1	0.5	0.25
Correct	27	27	33	27	21	28
Incorrect	19	19	13	13	19	12
A (in %)	4.74 (4.68)	4.15 (3.62)	3.52 (3.70)	7.70 (7.95)	5.43 (4.23)	5.59 (5.03)
B (in %)	5.37 (6.89)	5.59 (6.19)	5.43 (7.13)	7.43 (9.64)	5.65 (5.35)	6.93 (6.98)
Segments (in %)	37.0	24.4	11.5	65.1	45.1	25.6

The table shows results for simulated data not fulfilling the assumption of sequential tumor evolution. With increasing λ, an increasing number of samples are incorrectly classified. A) The average absolute difference between the estimated and true percentages in the simulated samples when the number of subpopulations are predicted correctly, B) The average absolute difference between the estimated and true percentages of the normal subpopulation. Standard deviations in parenthesis. 'Segments' is the number of segments violating the model of sequential tumor evolution.

### Validation experiment

The method was also validated using experimental data. We performed a series of hybridization experiments with different combinations of malignant and normal DNA as follows: 1) The tumor DNA is hybridized with normal DNA (pooled female healthy population). 2) A combination of 85% tumor DNA and 15% normal DNA is hybridized with normal DNA (pooled female healthy population). 3) A combination of 70% tumor DNA and 30% normal DNA is hybridized with normal DNA (pooled female healthy population). The hybridizations were performed using two different tumor DNA samples. For each of the hybridizations we estimated the number of subpopulations and percentages (Table 6). Since we do not know the composition of the tumor DNA, we do not know the true number of subpopulations and percentages, but based on our estimates we can predict what the estimated number of subpopulations and percentages should be in the mixed samples.

**Table 6:** Validation experiment

		Estimated based on (X = S1, S2)		
	Experiment	X	X with 15%	X with 30%
S1	76,24	-	79,21	77,23
S1 with 15%	82,18	80,20	-	81,19
S1 with 30%	84,16	83,17	85,15	-
S1	60,29,11	-	72,20,8	70,21,9
S1 with 15%	76,17,7	66,25,9	-	75,18,7
S1 with 30%	79,15,6	72,20,8	80,14,6	-
S2	62,24,14	-	49,33,18	50,31,19
S2 with 15%	57,28,15	68,20,12	-	57,27,16
S2 with 30%	65,22,13	73,17,10	65,23,12	-

In the "Experiment" column, the estimated subpopulation percentages from the 6 experiments are shown: Two pure tumor samples (S1 and S2), and four samples with tumor mixed with 15 or 30% normal cells. The best fit for S1 is three subpopulations, whereas it is two for "S1 with 15%" and "S1 with 30%"; therefore we show results for both two and three subpopulations to facilitate comparison. The remaining three columns contain percentages estimated from the Experiment column; e.g. to estimate the percentages of the sample with 85% malignant cells and 15% normal cells ("S2 with 15%") from the sample S2 do (0.62·85 + 15, 0.24·85, 0.14·85) = (68, 20, 12).

Table 6 shows that the predictions generally are in accordance with our expectations. All discrepancies between experimental and estimated percentages fall within the error bounds reported in Table 4. However, one of the tumor samples is best explained by three subpopulations (S1 in Table 6), whereas the two mixed samples ("S1 with 15%" and "S1 with 30%") are best explained by two subpopulations. By adding normal cells the signal from aberrant clones become diluted and it becomes more difficult to distinguish different abnormal subpopulations.

## Conclusion

Tumor heterogeneity is an important aspect of tumor evolution and progression. However, this aspect has, to the best of our knowledge, largely been ignored in analysis of CGH and SNP array data [12,13]. In Refs. [12,13], a fraction of the tumor is assumed to contain normal cells which weaken the signal from the aberrated cells. Only Ref. [13] estimates the fraction of normal cells directly, but we cannot compare this method to ours since it is developed to SNP array data. The method described in Ref. [12] does not output the frequency of normal cells. We have introduced a novel algorithm to estimate tumor heterogeneity and evaluated its performance on simulated and real tumor data. The method adds to our understanding of the genomic aberration profile, the quantification of genomic instability in the tumor, and the heterogeneity of the tumor.

One of the main difficulties of developing quantitative methods for array CGH data is the lack of knowledge about how tumors evolve and differentiate. Better and more accurate models could be developed if more were known about tumor evolution. Therefore, it might be difficult to make decisions on a strict mathematical basis only, because the underlying hypotheses might be difficult test or validate with current data sets. The appropriateness of the novel methodology can only be evaluated in a long run in which the conclusions demonstrate utility for improving biological understanding and clinical decisions. Our approach is one possible algorithm to interpret the biology of the tumor genomic profile.

In CGH array analysis copy number changes are measured relatively to a reference level. Generally, the reference level is not known and the median (or mean) log-intensity is typically assumed to correspond to two copies; loss and amplifications are then measured relatively to the median log-intensity level. Our method makes the same assumption. This implies that a tumor sample consisting of e.g. two subpopulations, one diploid and one *n*-ploid, would be identified as purely diploid. Each clone will have a log-intensity value that reflects the mixture of the two subpopulations and will, erroneously, be equated with two copies. However, if the two subpopulations are not euploid, our method might be able to disentangle the two subpopulations. This situation is not unlike traditional CGH array analysis where the tumor sample will be identified as one homogeneous population. Only if additional information is available, e.g. from karyotyping, can the reference level be properly adjusted.

We anticipate various lines of improvement, both in the chosen statistical methodology (e.g. to adopt a Bayesian framework to control the vast number of copy number parameters) as well in the mathematical modelling of tumor progression. These advances should be developed in tandem with richer and larger data set that are likely to occur with improved genomic technology. Our method (and improvements) can also be applied to SNP array data. Recent methods for SNP array analysis, e.g. [13,14], distingusih the two possible alleles; this might be useful for providing more accurate inference on copy numbers and the copy number level of the reference population, because each SNP carries two observations and not just one as for CGH arrays.

## Methods

### Materials

Cell lines with known copy number gains and losses were used to establish a copy number model. Here, we applied several cell lines including trisomy13, trisomy18, trisomy21 and 49, XXXXX. Normal male and female DNA were also used.

Twenty-nine pairs of primary breast tumors and their matched lymph node metastasis were provided by Copenhagen University Hospital. The project was approved by the Scientific and Ethical Committee of the Copenhagen and Frederiksberg Municipalities.

Arrays covering the whole genome with elements produced from bacterial artificial chromosome (BAC) clones were obtained from the Wellcome Trust Sanger Institute. The human DNA fragments of the 3340 BAC clones are spaced at approximately 1 Mb intervals across each chromosome arm. The experimental process is explained in details in [15]. Briefly, each clone is spotted on slides in a neighboring triplicate pattern. Annotations of the clones are based on the 1-Mb clone information published by the Wellcome Trust Sanger Institute and updated using the 38_36 version of the 1-Mb clone information released by Ensembl.

### Normalization of arrays

The intensities of Cy3 (tumor sample) and Cy5 (reference) were extracted respectively from 16 bit TIF files using the Tracker (Applied Precision) software. Subsequently data were subjected to quality assessment and a filtering process to remove the clones with poor quality.

Clones were removed from the subsequent analysis if one of the following conditions is fulfilled: a) The spot is labeled "Undetected" by Tracker, b) The Sanger annotation of a clone is inconsistent with the Ensemble annotation (see above), c) The spot's Cy5 (reference) intensity is less than two times the standard deviation (SD) of its background intensity, d) Only one spot out of the three replicates is left after the above procedure, e) The CV of the intensity ratios Cy3/Cy5 for one clone exceeds 0.08, and f) The clone maps to chromosome Y.

Finally, the ratios of Cy3/Cy5 intensities are calculated and log transformed. Subsequently, the median of the log-ratios from the whole array is subtracted from each log-ratio to normalize all spots.

### The copy number model

We modeled the Cy3/Cy5 intensity ratios in the following way. Assume that the test sample is homogeneous, i.e. a given clone has the same copy number in all cells in the sample, and that the clones are divided into distinct segments such that all clones in a segment have the same copy number.

Let *x*_*ij *_be the ratio of clone *j *in segment *i*, and let *C*_0*i *_and *C*_1*i *_be the copy numbers of the reference sample and the test sample, respectively. We assume

(3) $$x_{ij}=\gamma {\left(\frac{C_{1i}}{C_{0i}}\right)}^{\alpha }(1+\epsilon_{ij}),$$

where *γ *is a constant depending on the quality of the DNA in the tube, amplification, scanning and other hybridization and experimental conditions. The error term *ε*_*ij *_is assumed to have mean zero and common variance, and *α *is a constant that is justified from calibration experiments [see Additional file 1] and appears to be sample independent (but likely platform specific). The model assumes the variance is proportional to the true ratio of copy numbers in the test and reference samples.

Let *x*_*R *_be the median over all intensity ratios (over all segments). Then

(4) $$z_{ij}=\frac{x_{ij}}{x_{R}}={\left(\frac{C_{1i}C_{0R}}{C_{1R}C_{0i}}\right)}^{\alpha }(1+{\epsilon^{\prime }}_{ij}),$$

where *C*_1*R *_(*C*_0*R*_) is the copy number of the test (reference) sample corresponding to *x*_*R *_and *ε' *is an error term (not equal to *ε*). The error term is defined such that (1 + *ε*_*ij*_)/(1 + *ε*_*R*_) = 1 + ${\epsilon^{\prime }}_{ij}$. Typically, the majority of clones in a tumor sample have copy number two and we assume *C*_1*R *_= 2. In general, *C*_0*i*_/*C*_0*R *_= 1 in the reference sample, unless the reference sample has only one chromosome X and *C*_0*i*_/*C*_0*R *_= 1/2 for chromosome X clones.

Put *C*_*i *_= *C*_1*i*_/*C*_1*R*_, *C*_*i *_∈ {0, $\frac{1}{2}$, 1, $\frac{3}{2}$, ...}, then *z*_*ij *_= $C_{i}^{\alpha }$(1 + ${\epsilon^{\prime }}_{ij}$). We refer to *z*_*ij *_as the normalized (intensity) ratio. With this notation we have

(5) $$\begin{array}{lll} \log(z_{ij}) & = & \alpha \log(C_{i})+\log(1+{\epsilon^{\prime }}_{ij})\\ & \approx & \alpha \log(C_{i})+{\epsilon^{\prime }}_{ij}. \end{array}$$

Further, assume

(6) log(*z*_*ij*_) ~ *N*(*α*log(*C*_*i*_) + *β*, *σ*^2^),

where *β *is the mean of ${\epsilon^{\prime }}_{ij}$ and *σ*^2 ^the variance. Equation (3) ensures the variance is independent of the copy number. If a series of experiments with known copy numbers are available, the parameters in equation (6) can be estimated using linear regression.

In order to determine *α *and *β *in equation (6), we used the normal references and the samples with known copy number aberrations. We have data corresponding to the following ratios: 0.5 (46, XY versus 46, XX), 1.5 (47, XX+13 versus the normal reference, and 47, XX+18 versus the normal reference), 2 (chr X, the normal females versus the normal males), and 2.5 (49, XXXXX versus 46, XX).

### Mixture modeling of tumor samples

The intensity ratios in a tumor sample is modeled using a mixture model approach. Specifically, the log-ratio log(*z*_*ij*_) has intensity given by

(7) $$\log(z_{ij})\sim N(\alpha \log({\bar{C}}_{i})+\beta ,\sigma^{2}),$$

where

(8) $${\bar{C}}_{i}=\sum_{k=0}^{K}p_{k}C_{ik},$$

*K *is the number of subpopulations, *p*_*k *_is the percentage of the *k*th subpopulation, Σ_*k*_*p*_*k *_= 1, and *C*_*ik *_≥ 0 is the copy number in the *k*th subpopulation relative to the copy number of the test sample. Normally, the same region in different subpopulations will not experience both gains and losses [16].

Therefore, we restrict our model parameters in the following way. The first subpopulation, with percentage *p*_0_, is assumed to be normal; i.e. *C*_*i*0 _= 1 for all clones in this subpopulation. We assume the other subpopulations are derived from each other, such that either

(9) 1 = *C*_*i*0 _≤ *C*_*ik *_≤ *C*_*i*,*k*+1_

or

(10) 1 = *C*_*i*0 _≥ *C*_*ik *_≥ *C*_*i*,*k*+1_.

That is, we consider subpopulation *k *+ 1 to be derived from subpopulation *k *by either A) introducing a new copy number aberration (*C*_*ik *_= 1, but *C*_*i*,*k*+1 _≠ 1), B) increasing an existing copy number gain, or C) increasing an existing copy number loss.

### Estimation of copy numbers and percentages

To estimate the copy numbers and the percentages of the subpopulations, we first divide the clones into segments, such that all clones in a segment have the same copy number profile. To segment the clones, we used DNAcopy [17] implemented in R. A comparison study of several segmentation approaches have been done recently [18], and DNAcopy came out best.

After segmentation, all clones in one segment are assigned the same value, namely the mean of the intensity values in that particular segment. Missing clone values mapping within a segment are given the same value as the segment, while missing clone values located between segments have values imputed using the minimum absolute value of the two flanking segments. The copy number level closest to zero is declared unchanged ("normal level") and corresponds to two copies. In the final step, all segments are normalized by subtracting the value of the normal level.

Denote by ${\hat{\sigma }}^{2}$ the residual error

(11) $${\hat{\sigma }}^{2}=\frac{1}{M}\sum_{ij}{\left(\log(z_{ij})-{\hat{\mu }}_{j}\right)}^{2}$$

where *M *is the total number of clones, *m*_*i *_the number of clones in segment *i*, and

$${\hat{\mu }}_{i}=\frac{1}{m_{i}}\sum_{j}\log(z_{ij})$$

the mean intensity of segment *i*.

For a given number of subpopulations, *K *= 2, 3, or 4, we use least square to fit the parameters (copy numbers, percentages); i.e. for each *K *we minimize

(12) $$LS=\sum_{i,j}{\left(\log(z_{ij})-\hat{\alpha }\log({\bar{C}}_{i})-\hat{\beta }\right)}^{2}$$

over *p*_*k*_, *k *= 0, ..., *K *and *C*_*ik *_with the constraints given in equations (9) and (10). Here ($\hat{\alpha }$, $\hat{\beta }$) is obtained from the samples with known copy number alterations and assumed to be known in equation (12).

Alternatively, one can minimize

(13) $$LS^{\prime }=\sum_{i}m_{i}{\left({\hat{\mu }}_{i}-\hat{\alpha }\log({\bar{C}}_{i})-\hat{\beta }\right)}^{2},$$

where *m*_*i *_is the number of clones in segment *i*. Equation (13) involves summation over fewer terms than equation (12) and might thus be preferred. For fixed *K*, there are *K *- 1 percentage parameters and *K *- 1 copy numbers for each segment; in total (*K *- 1) + *n*(*K *- 1) = (*n *+ 1)(*K *- 1) parameters, where *n *is the number of segments. The number of parameters scales with the number of clones; however, since the copy numbers assume integer values we do not obtain a perfect fit to the log-intensities in equation (12) or (13). To facilitate comparison between different experiments, we use the normalized quantity $NLS=\sqrt{LS^{\prime }/n}$, where *n *is the number of segments in one experiment.

### Classification of samples

To classify a sample we go through the following steps. The estimation procedure outlined in the previous section is applied.

#### Estimation of subpopulation number and parameters

1) Apply DNACopy to obtain a list of segments.

2) Fit *K *subpopulations to obtain *NLS*_*K*_, *K *= 2, 3, 4, with corresponding percentages (*p*_0_, *p*_1_, ..., *p*_*K*_) and copy number profiles (*C*_*i*1_, ..., *C*_*iK*_). The first subpopulation is supposed to consist of pure normal cells.

#### Simulation of bootstrap samples

To simulate bootstrap samples the estimated copy number profiles are applied. Noise are added to the profiles to obtain log-intensity values.

3) Choose *α *and *β *according to the estimated normal distributions obtained by linear regression. The distributions are restricted to the 95% CI to avoid extreme values.

4) For fixed *K*, simulate log-intensity values for each estimated copy number profile, *k *= 1, ..., *K*, by adding noise: For a clone with copy number *C*, compute the mean log-intensity *β *+ *αC *and add noise according to a normal distribution *N*(0, ${\hat{\sigma }}^{2}=\frac{1}{M}\sum_{ij}{\left(\log(z_{ij})-{\hat{\mu }}_{j}\right)}^{2}$).

5) Repeat the previous step *B *times for each sample and each value of *K *to obtain simulated samples with *K *= 2, 3 or 4 subpopulations. For each simulated sample fit 2, 3 and 4 subpopulations according to step 1 and 2, and calculate the corresponding *NLS*_*bKC*_. Here *b *denotes the *b*th simulated/bootstrapped sample with *K *subpopulations, fitted to *C *subpopulations, *C *= 2, 3, 4.

#### Evaluation of NLS_K _from real samples

In the final step the *NLS*_*K*_, *K *= 2, 3, 4, from a real sample is compared to the bootstrapped samples to find the optimal number of subpopulations for the real sample.

6) If *NLS*_2 _is below the 95 percentile of the empirical distribution of *NLS*_*b*22_, accept the sample as two populations, otherwise

7) If *NLS*_3 _is below the 95 percentile of the empirical distribution of *NLS*_*b*33_, accept the sample as three populations, otherwise

8) Accept the sample as 4 subpopulations.

**Figure 3:**
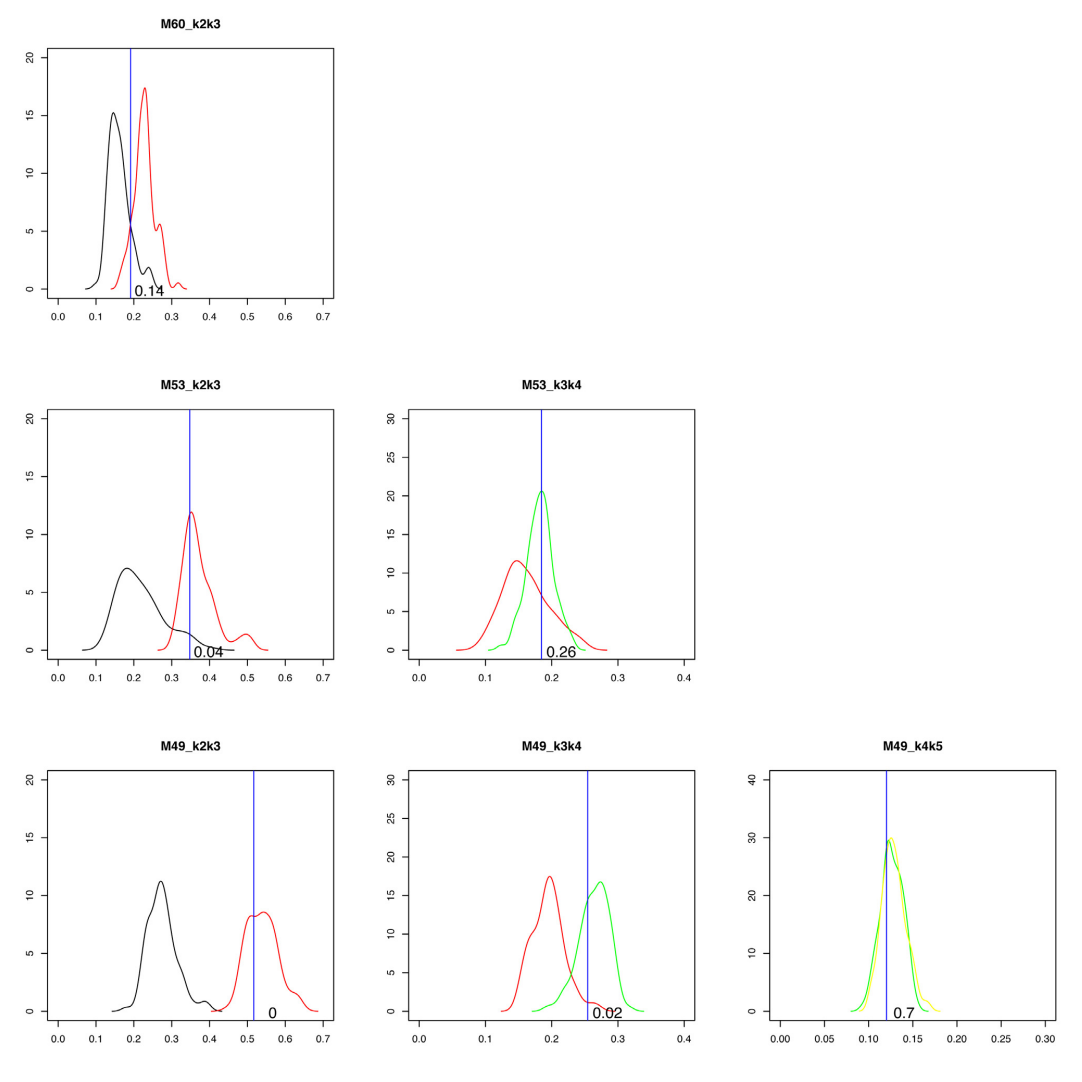
**Classifier**. Here we show three examples of classification of real samples. From top to bottom, the three samples are classified as 2, 3, and 4 subpopulations, respectively. Each subplot shows two empirical distributions and a blue line representing the *NLS*_*K *_of the query sample. In the first column, the black curve is the smoothed empirical distribution (SED) of *NLS*_*b*22 _(simulated as two subpopulations and fitted as two) and the red curve is the SED of *NLS*_*b*32 _(simulated as three and fitted as two). In the second column, the red curve is the SED of *NLS*_*b*33 _(simulated as three and fitted as three) and the green curve is the SED of *NLS*_*b*43 _(simulated as four and fitted as three). Finally, in the last column, the green curve is the SED of *NLS*_*b*44 _(simulated as four and fitted as four) and the yellow curve is the SED of *NLS*_*b*54 _(simulated as five and fitted as four). The number in the subplots shows how many samples (in %) in the left distribution that obtain a value greater than the value indicated by the blue line.

The part described in steps 6–8 is illustrated in Figure 3. The whole procedure is a bootstrap procedure; for a real sample the fitted profiles (one for each subpopulation) are compared to simulated samples with the same profiles as the fitted. For a (supposedly) normal sample, one can start with a single population, *K *= 1 (only normal cells).

### Simulation

To test the classifier we choose some of the simulated samples and used these as input to the bootstrap procedure described above. For each real sample we choose four simulated samples as input and compared the result to the known input.

We also tested how robust the classifier is to deviations from the assumption of sequential tumor evolution. This we did by adding or subtracting a Poisson number of copies to the original copy number. For each segment, *S*_*i*_*X*_*i *_copies were added to the original copy number in subpopulation *i*. Here, *P*(*S*_*i *_= 1) = *P*(*S*_*i *_= 1) = 0.5 and *X*_*i *_is Poisson(*λ*). If the copy number fell below 0, it was put to zero. The parameter *λ *was varied over *λ *= 0.25, 0.5 and 1. For each real sample with *K *= 3 estimated subpopulations we simulated 2 samples (in total 2·23 = 46 simulated samples) in this way, and for each real sample with *K *= 4 we simulated 4 samples (in total 4·10 = 40 simulated samples).

## Authors' contributions

KW and CW developed the method with input from the other authors, KW and SL implemented the method, KW and JL carried out the data analysis, and KW and CW wrote the manuscript with input from JL and LB; JL performed all experiments. All authors read and approved the final manuscript.

## Supplementary Material

Additional file 1 **Regression analysis.** The figure shows the the observed averaged intensity values from clones with known copy number changes (e.g. trisomies) and the linear regression fit to the observed values. The x-axis represents the known log_2 _copy number ratio (copy number divided by 2) and the y-axis represents the observed log_2 _intensity ratio. The blue spots represent the observed averaged intensities and red spots show the predicted values. Click here for file

Additional file 2 **Detailed results for the 29 pairs of primary tumors and lymph node metastasis.** The table shows results for the 29 pairs of tumors organized in two times eight columns. ID: Name of sample, Pops: Estimated number of subpopulations, %: Subpopulation percentages, *AI*_*k*_: Aberration Index for subpopulation *k*, excluding the normal subpopulation, Total: Weighted sum of *AI*_*k*_, Σ_*k*_*p*_*k*_*AI*_*k*_, Pure: *AI*_•_, normalized weighted sum of *AI*_*k*_, Σ_*k*_*p*_*k*_*AI*_*k*_/(1 - *p*_0_). Click here for file
