# Supplementary figures and images for: Estimation of tumor heterogeneity using CGH array data
Source: BMC Bioinformatics. 2009 Jan 9;10:12. doi: 10.1186/1471-2105-10-12 (PMC2640360; doi:10.1186/1471-2105-10-12)

# Copy Number Model

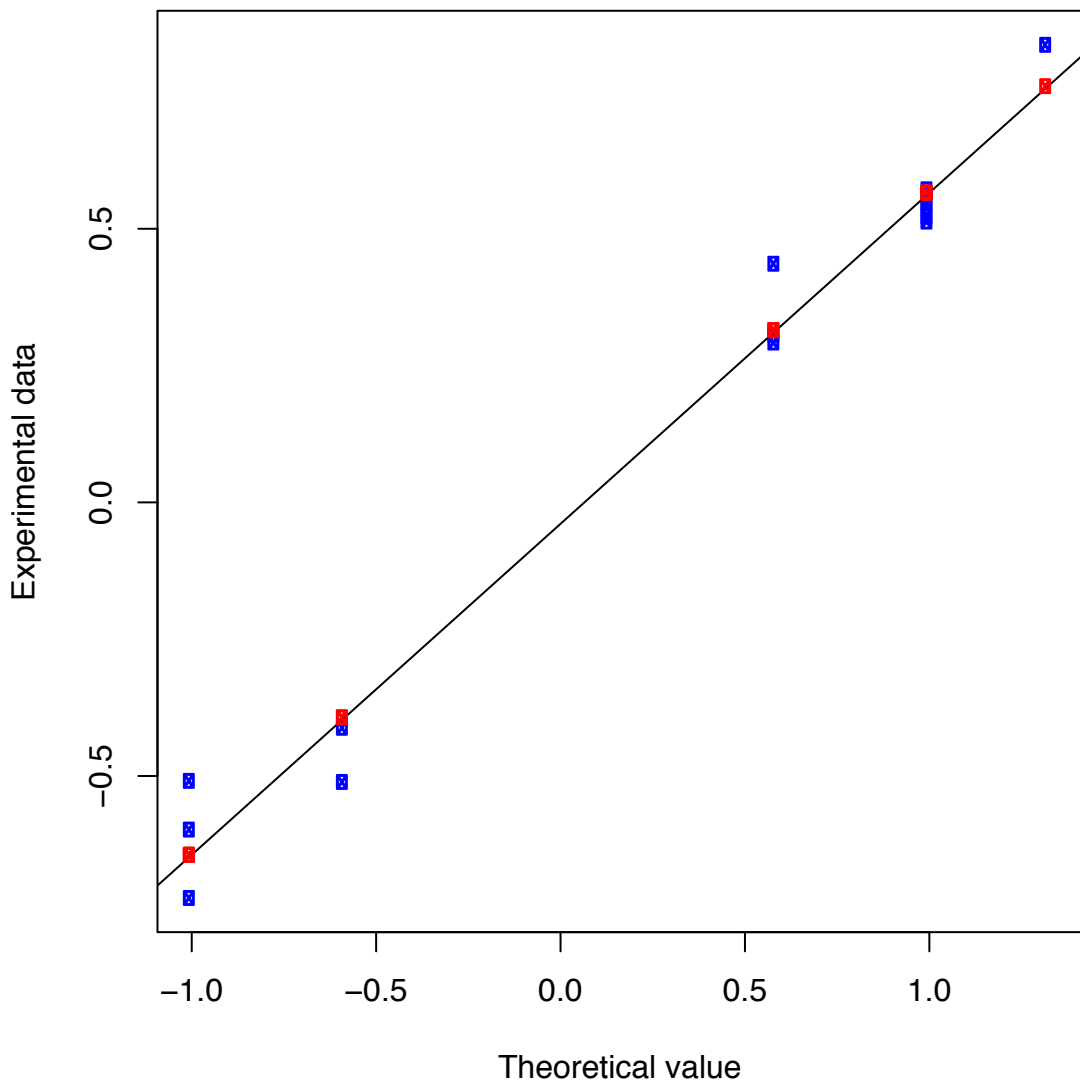

Supplement: Additional file 1 — Regression analysis. The figure shows the the observed averaged intensity values from clones with known copy number changes (e.g. trisomies) and the linear regression fit to the observed values. The x-axis represents the known log2 copy number ratio (copy number divided by 2) and the y-axis represents the observed log2 intensity ratio. The blue spots represent the observed averaged intensities and red spots show the predicted values. [file 1471-2105-10-12-S1.pdf]
