# Supplementary material for: Estimation of tumor heterogeneity using CGH array data
Source: BMC Bioinformatics. 2009 Jan 9;10:12. doi: 10.1186/1471-2105-10-12 (PMC2640360; doi:10.1186/1471-2105-10-12)
Supplement: Additional file 2 — Detailed results for the 29 pairs of primary tumors and lymph node metastasis. The table shows results for the 29 pairs of tumors organized in two times eight columns. ID: Name of sample, Pops: Estimated number of subpopulations, %: Subpopulation percentages, AIk: Aberration Index for subpopulation k, excluding the normal subpopulation, Total: Weighted sum of AIk, ΣkpkAIk, Pure: AI•, normalized weighted sum of AIk, ΣkpkAIk/(1 - p0). [file 1471-2105-10-12-S2.pdf]

ID	Pops	%	AI_1	AI_2	AI_3	Total	Pure	ID	Pops	%	AI_1	AI_2	AI_3	Total	Pure
T47	4	35,23,34,8	0.25	0.53	1.40	0.35	0.54	M47	3	42,40,18	0.38	1.02	-	0.33	0.58
T49	2	78,22	0.71	-	-	0.16	0.71	M49	4	32,20,37,11	0.14	0.36	1.22	0.29	0.43
T50	2	70,30	1.73	-	-	0.52	1.73	M50	2	67,33	1.00	-	-	0.33	1.00
T51	3	52,19,29	0.46	1.37	-	0.49	1.01	M51	3	52,18,30	0.56	1.19	-	0.46	0.95
T53	2	51,49	0.21	-	-	0.10	0.21	M53	3	48,33,19	0.23	0.50	-	0.17	0.33
T55	2	66,34	0.69	-	-	0.23	0.69	M55	2	80,20	0.60	-	-	0.12	0.60
T57	2	78,22	1.11	-	-	0.24	1.11	M57	3	56,28,16	0.76	1.73	-	0.49	1.11
T58	2	78,22	0.99	-	-	0.22	0.99	M58	3	63,24,13	0.43	1.48	-	0.30	0.80
T60	2	86,14	1.67	-	-	0.23	1.67	M60	2	76,24	1.56	-	-	0.37	1.56
T61	2	74,26	1.16	-	-	0.30	1.16	M61	3	63,26,11	0.85	1.93	-	0.43	1.17
T64	3	58,34,8	0.22	1.04	-	0.16	0.38	M64	2	79,21	0.72	-	-	0.15	0.72
T69	3	52,36,12	0.59	1.75	-	0.42	0.88	M69	2	65,35	0.52	-	-	0.18	0.52
T70	2	74,26	1.49	-	-	0.39	1.49	M70	4	45,16,31,8	0.19	0.58	1.52	0.33	0.60
T74	3	49,37,14	0.47	1.30	-	0.36	0.70	M74	2	76,24	1.47	-	-	0.35	1.47
T75	2	82,18	0.79	-	-	0.14	0.79	M75	2	69,31	0.45	-	-	0.14	0.45
T77	3	31,54,15	0.38	1.17	-	0.38	0.55	M77	3	55,33,12	0.54	1.35	-	0.34	0.75
T78	2	81,19	0.50	-	-	0.09	0.50	M78	2	74,26	0.62	-	-	0.16	0.62
T82	2	71,29	0.30	-	-	0.09	0.30	M82	2	87,13	1.06	-	-	0.14	1.06
T83	3	55,32,13	0.24	1.43	-	0.26	0.58	M83	2	66,34	0.25	-	-	0.09	0.25
T84	3	44,38,18	0.18	0.75	-	0.20	0.36	M84	4	54,14,25,7	0.21	0.50	1.25	0.24	0.52
T85	3	40,44,16	0.22	1.12	-	0.28	0.46	M85	3	51,39,10	0.09	1.37	-	0.17	0.35
T86	2	75,25	1.55	-	-	0.39	1.55	M86	2	86,14	2.72	-	-	0.38	2.72
T88	3	54,34,12	0.32	1.13	-	0.24	0.53	M88	3	53,37,10	0.27	1.10	-	0.21	0.45
T90	3	68,21,11	0.46	1.35	-	0.25	0.77	M90	2	84,16	0.70	-	-	0.11	0.70
T91	3	53,33,14	0.15	0.91	-	0.18	0.38	M91	2	66,34	0.40	-	-	0.14	0.40
T93	2	86,14	3.27	-	-	0.46	3.27	M93	2	90,10	4.04	-	-	0.40	4.04
T94	3	63,26,11	0.32	1.05	-	0.20	0.54	M94	3	41,47,12	0.39	1.68	-	0.39	0.66
T95	2	70,30	0.71	-	-	0.21	0.71	M95	2	70,30	0.53	-	-	0.16	0.53
T98	3	62,26,12	0.35	0.99	-	0.21	0.55	M98	2	69,31	0.45	-	-	0.14	0.45
